# Lemmel Syndrome Secondary to Duodenal Diverticulitis: A Case Report

**DOI:** 10.7759/cureus.1066

**Published:** 2017-03-01

**Authors:** Keyur Desai, Joshua D Wermers, Nebiyu Beteselassie

**Affiliations:** 1 Department of Radiology, Truman Medical Center; 2 Department of Radiology, UMKC

**Keywords:** lemmel syndrome, duodenal diverticulum, duodenal diverticulitis, mrcp, ct

## Abstract

Lemmel syndrome occurs when a duodenal diverticulum causes obstructive jaundice due to a mechanical obstruction of the common bile duct. Additional pathophysiologic processes may also contribute to the development of Lemmel syndrome. These include duodenal diverticula causing dysfunction of the sphincter of Oddi as well as compression of the common bile duct by duodenal diverticula. It is uncommon for duodenal diverticulum to become inflamed. We report the case of a 25-year-old female presenting with unintentional weight loss and fatigue. Since her initial labs were concerning for possible infection with hepatobiliary abnormalities, a contrast-enhanced CT was obtained. This study revealed a large periampullary diverticulum with mucosal enhancement and fat stranding consistent with diverticulitis.

## Introduction

Duodenal diverticula are most often found in the second part of the duodenum adjacent to the ampulla of Vater. These diverticula are pseudo-diverticula consisting of outpouchings of mucosa, which lack a muscularis layer. When these diverticula are located within 2-3 cm of the ampulla of Vater they are termed periampullary diverticula [1]. Periampullary diverticula are usually asymptomatic but, in rare instances, can cause pancreaticobiliary complications when inflamed [2]. Obstructive jaundice can develop secondary to periampullary diverticula without choledocholithiasis in the setting of Lemmel syndrome [3]. We present a case of Lemmel syndrome secondary to duodenal diverticulitis that was successfully managed with nasogastric (NG) tube decompression set to suction as well as intravenous antibiotics, including metronidazole and levofloxacin. Informed patient consent was obtained from this patient.

## Case presentation

**Figure 1:**
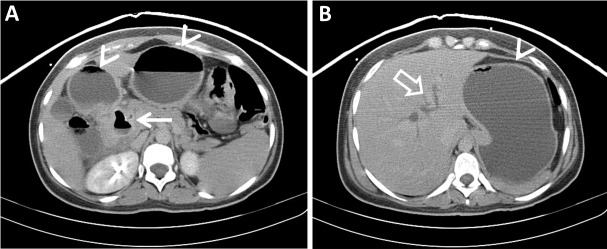
Contrast-enhanced Axial CT of the Upper Abdomen (Pre-Treatment) Contrast-enhanced axial CT images of the upper abdomen demonstrate (A) a periampullary duodenal diverticulum with surrounding inflammatory changes consistent with diverticulitis, including wall thickening and fat stranding (arrow). (B) An axial slice slightly more cephalad shows dilated intrahepatic bile ducts (open arrow). Also seen are the stomach and proximal duodenum, which are dilated with air-fluid levels (arrowheads).

**Figure 2:**
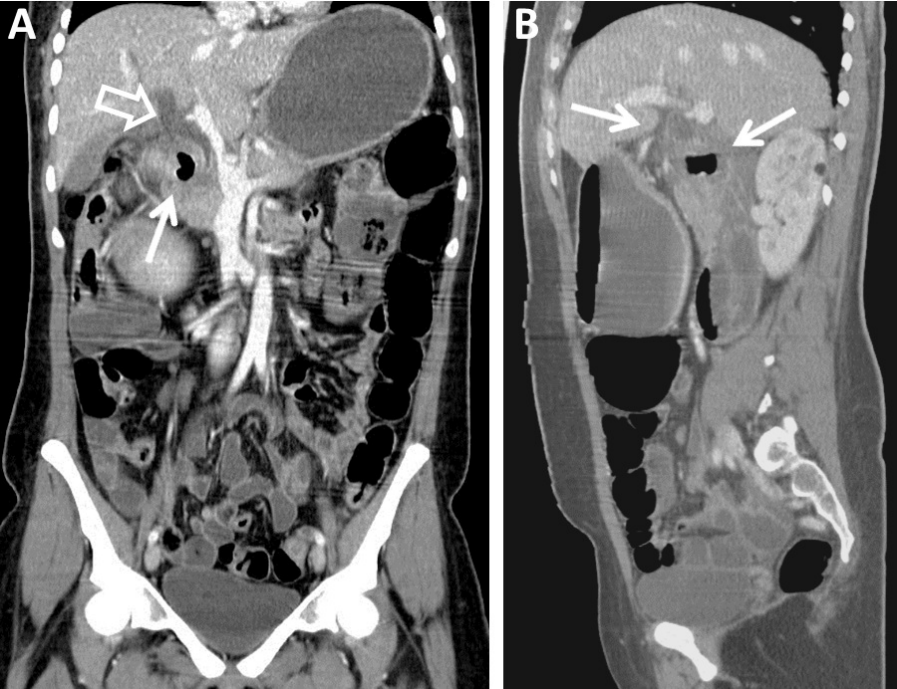
Contrast-enhanced Coronal and Sagittal CT of the Abdomen and Pelvis (Pre-Treatment) Contrast-enhanced reformatted images of the abdomen and pelvis demonstrate (A) an inflamed periampullary diverticulum, which obstructs the common bile duct (arrow). The common bile duct (open arrow) is significantly dilated. In addition, intrahepatic biliary ductal dilation is seen. (B) The sagittal image provides another view of the duodenal diverticulitis and common bile duct dilation (arrows).

A 25-year-old previously healthy woman presented to the emergency department without any significant past medical history. She had a 30-pound unintentional weight loss, fatigue, and weakness that began three months prior. The patient denied any abdominal pain, fever, chills, nausea, vomiting, melena, hematochezia, and hematemesis. Her entire physical examination was negative, including the absence of jaundice. Laboratory values revealed that hemoglobin and bilirubin metabolites were normal, but inflammatory markers, including CRP, were elevated. In addition, alkaline phosphatase, serum aspartate aminotransferase (AST), alanine transaminase (ALT), and white cell count were all elevated. The remaining laboratory values were unremarkable. Contrast-enhanced computed tomography (CT) of the abdomen and pelvis was obtained and revealed a large periampullary diverticulum containing a fluid level with wall thickening, mucosal enhancement, and surrounding fat stranding consistent with duodenal diverticulitis. The diverticulum appeared to cause common bile duct compression with resultant upstream dilation of intrahepatic biliary ducts (Figures 1-2).

**Figure 3:**
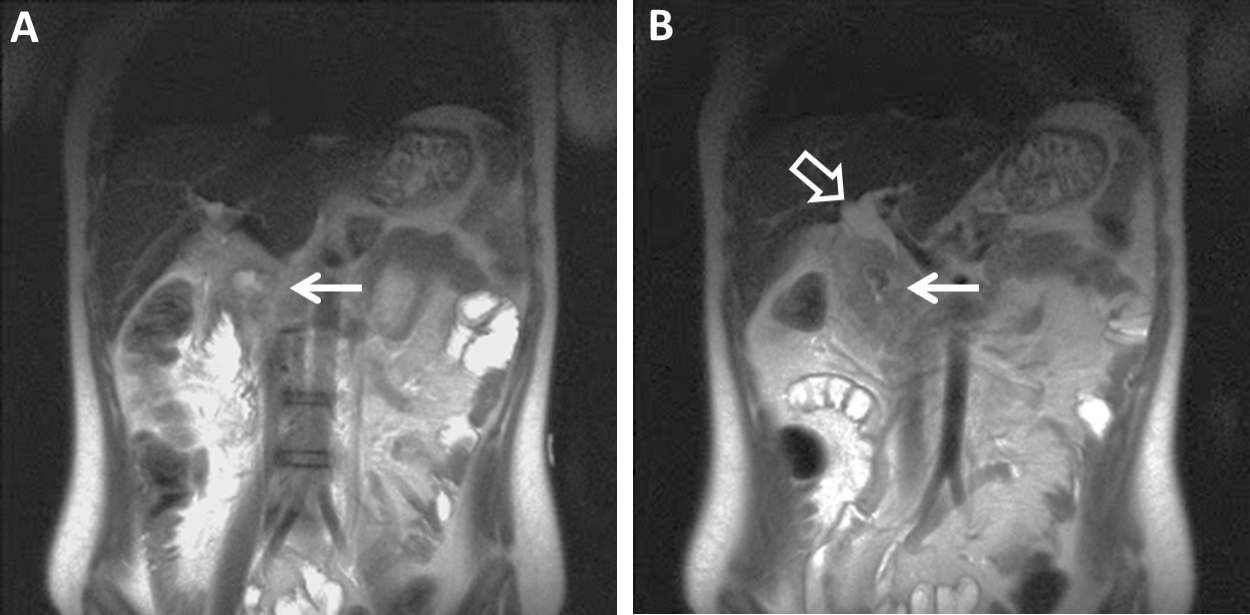
T2-weighted Coronal MRI of the Abdomen (Pre-Treatment) T2-weighted coronal MR images of the abdomen re-demonstrate (A) a periampullary duodenal diverticulum with surrounding inflammatory changes (arrow).  (B) A more posterior slice demonstrates a dilated common bile duct and proximal intrahepatic ducts (open arrow).

In addition, the stomach and proximal duodenum were dilated with air-fluid levels concerning for proximal obstruction. Surgery was consulted and decided immediate surgical intervention was not warranted. Surgery did have some suspicion that the patient may have an underlying malignancy and magnetic resonance imaging/magnetic resonance cholangiopancreatography (MRI/MRCP) was obtained to better characterize the biliary ductal dilation. This re-demonstrated a periampullary duodenal diverticulum with adjacent inflammatory changes. The inflamed diverticulum caused a mass effect of the distal intrapancreatic common bile duct with resultant dilatation of the proximal common bile duct measuring up to 1.8 cm. Mild intrahepatic biliary ductal dilation was once again seen (Figure 3).

**Figure 4:**
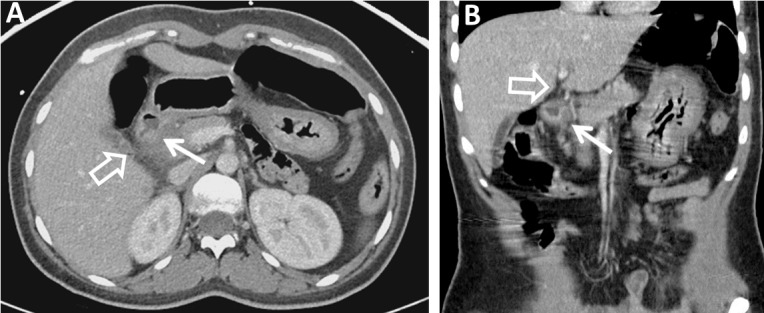
Contrast-enhanced Axial (A) and Coronal (B) CT Images of the Abdomen and Pelvis (Post-Treatment) Contrast-enhanced axial (A) and coronal (B) CT images of the abdomen and pelvis two weeks after conservative treatment demonstrate a small duodenal diverticulum with significantly improved surrounding inflammatory changes (arrows). Intrahepatic biliary ductal dilation is significantly improved (open arrow).

Otherwise, the other abdominal organs were normal. The patient was subsequently made nil per os (NPO) and an NG tube was placed for decompression and suction. She completed a 14-day course of intravenous antibiotics, including metronidazole and levofloxacin. Follow-up CT two weeks later revealed a significant decrease in the inflammation, and the patient tolerated advancing diet (Figure 4).

Surgery considerations were postponed at this point in time due to reduction of inflammation and asymptomatic patient. Follow-up laboratory values revealed normalization of the previously elevated alkaline phosphatase, AST, and ALT levels.

## Discussion

Gastrointestinal diverticula are outpouchings of the intestinal wall, which can occur anywhere throughout the gastrointestinal tract and are most often found in the colon, followed by the duodenum. Duodenal diverticula are classified based on their location. Amongst the different types of duodenal diverticula, periampullary duodenal diverticula are the most common [4]. Although the majority of periampullary diverticula are asymptomatic, occasionally non-pancreaticobiliary or pancreaticobiliary complications can occur. Non-pancreaticobiliary complications are rare and may include diverticulitis, hemorrhage, perforation, or fistula formation. Pancreaticobiliary complications can present as recurrent gallbladder or bile duct stones, obstructive jaundice, cholangitis, or acute pancreatitis [5].

Lemmel syndrome was first described in 1934 by Lemmel as obstructive jaundice in the absence of gallstones due to a periampullary duodenal diverticulum. This can be recurrent or complicated by cholangitis, which is attributed to mechanical compression of the terminal bile duct by diverticulum [6]. There are various etiologies regarding the pathogenesis leading to the development of Lemmel syndrome. First, direct mechanical irritation of periampullary diverticula may cause chronic inflammation of the ampulla, which leads to fibrosis of the papilla. Second, periampullary diverticula may cause dysfunction of the sphincter of Oddi. Third, the distal common bile duct or ampulla can be compressed mechanically by periampullary diverticula as occurs in our case [7].

Imaging is very important to correctly identify and diagnose Lemmel syndrome, and awareness of this condition may prevent mismanagement. Using CT scan and MRCP, periampullary diverticula may appear as thin-walled cavitary lesions on the medial wall of the second portion of the duodenum. Sometimes these diverticula can be filled with fluid and misdiagnosed as a pancreatic abscess, cystic neoplasm in the pancreatic head, or as a metastatic lymph node [8]. Barium studies can demonstrate this condition with contrast-filled outpouchings that arise from the medial side of the descending duodenum [9].

Regarding treatment, surgical excision of the diverticulum is appropriate in certain clinical scenarios. When a biliary obstruction is present, excision of the diverticulum can be done, but the procedure is difficult and associated with significant mortality [6]. In relatively asymptomatic patients, non-surgical or conservative treatment is recommended. However, many patients with Lemmel syndrome do present with some form of symptoms related to biliary obstruction secondary to extrinsic compression of the common bile duct and treatment may be warranted. Various management options exist ranging from endoscopic extraction of entrapped material, extracorporeal shock wave lithotripsy, to laparoscopic diverticulectomy [10]. Another important consideration is that not all forms of Lemmel syndrome can be attributed to extrinsic compression of the common bile duct (CBD) by periampullary diverticula. Depending on the underlying mechanism and pathogenesis, treatment modality may vary.

## Conclusions

Lemmel syndrome is a rare cause of biliary obstruction. It is an obstructive jaundice in the absence of gallstones primarily due to a periampullary duodenal diverticulum. Principal etiologies regarding pathogenesis in developing Lemmel syndrome include direct mechanical irritation of periampullary diverticula, dysfunction of the sphincter of Oddi, and mechanical compression of the distal common bile duct. Imaging is critical in diagnosing Lemmel syndrome. CT scan and MRCP will demonstrate periampullary diverticula on the medial wall of the second portion of the duodenum. Treatment modality of Lemmel syndrome may vary depending on the underlying mechanism and pathogenesis. Extrinsic compression of the common bile duct may require surgery. In our case, surgery was considered but ultimately was not needed due to the resolution of inflammation on follow-up CT imaging with a 14-day course of intravenous antibiotics. Follow-up laboratory values revealed normalization of previously elevated alkaline phosphatase, AST, and ALT levels.
